# Spatial distribution, magnitude, and predictors of high fertility status among reproductive age women in Ethiopia: Further analysis of 2016 Ethiopia Demographic and Health Survey

**DOI:** 10.1371/journal.pone.0290960

**Published:** 2023-09-08

**Authors:** Desale Bihonegn Asmamaw, Wubshet Debebe Negash, Fantu Mamo Aragaw, Daniel Gashaneh Belay, Melaku Hunie Asratie, Abel Endawkie, Tadele Biresaw Belachew

**Affiliations:** 1 Department of Reproductive Health, Institute of Public Health, College of Medicine and Health Sciences, University of Gondar, Gondar, Ethiopia; 2 Department of Health Systems and Policy, Institute of Public Health, College of Medicine and Health Sciences, University of Gondar, Gondar, Ethiopia; 3 Department of Epidemiology and Biostatistics, Institute of Public Health, College of Medicine and Health Sciences, University of Gondar, Gondar, Ethiopia; 4 Department of Human Anatomy, College of Medicine and Health Sciences, University of Gondar, Gondar, Ethiopia; 5 Department of Women’s and Family Health, School of Midwifery, College of Medicine and Health Sciences, University of Gondar, Gondar, Ethiopia; 6 Department of Epidemiology and Biostatistics, School of Public Health, College of Medicine and Health Science, Wollo University, Dessie, Ethiopia; Debre Berhan University, ETHIOPIA

## Abstract

**Background:**

Women’s health and welfare, as well as the survival of their children, are adversely affected by high fertility rates in developing countries. The fertility rate in Ethiopia has been high for a long time, with some pockets still showing poor improvement. Thus, the current study is aimed to assess the spatial distribution and its predictors of high fertility status in Ethiopia.

**Methods:**

Secondary data analysis was used using the 2016 Ethiopian Demographic and Health Survey (EDHS). The Bernoulli model was used by applying Kulldorff methods using the SaTScan software to analyze the purely spatial clusters of high fertility status. ArcGIS version 10.8 was used to visualize the distribution of high fertility status across the country. Mixed-effect logistic regression analysis was also used to identify the predictors of high fertility.

**Result:**

High fertility among reproductive-age women had spatial variation across the country. In this study, a higher proportion of fertility occurred in Somali region, Southeastern part of Oromia region, and Northeastern part of SNNPR. About 45.33% (confidence interval: (44.32, 46.33) of reproductive-age women had high fertility. Education; no formal (aOR: 13.12, 95% CI: 9.27, 18.58) and primary (aOR: 5.51, 95% CI: 3.88, 7.79), religion; Muslim (aOR: 1.52, 95% CI: 1.28, 1.81) and Protestant (aOR: 1.48, 95% CI: 1.23, 1.78), age at first birth (aOR: 2.94, 95% CI: 2.61, 3.31), age at first sex (aOR: 1.70, 95% CI: 1.49, 1.93), rural resident (aOR: 3.76, 95% CI: 2.85, 4.94) were predictors of high fertility in Ethiopia.

**Conclusion:**

The spatial pattern of high fertility status in Ethiopia is clustered. Hotspot areas of a problem were located in Somali, Central Afar, Northeastern part of SNNPR, and Southeastern part of Oromia region. Therefore, designing a hotspot area-based interventional plan could help to reduce high fertility. Moreover, much is needed to be done among rural residents, reducing early sexual initiations and early age at first birth, and enhancing women’s education. All the concerned bodies including the kebele administration, religious leaders, and community leaders should be in a position to ensure the practicability of the legal age of marriage.

## Introduction

Women with high fertility are those who have at least five pregnancies at gestational ages greater than or equal to 20 weeks over their reproductive period, which is a major public health problem in developing countries, particularly in sub-Saharan Africa and Ethiopia [1,2]. It has negative health consequences for children and their mothers, slows economic growth, and exacerbates environmental problems [3].

Women with five or more pregnancies are at an increased risk for maternal death [4]. In addition, high-fertility countries have poor child survival rates. For instance, for every 100,000 births in African countries with high fertility, 640 women die during pregnancy and childbirth [5]. In Sub-Saharan Africa, population growth is outpacing economic growth. Many countries have fertility rates that are far higher than replacement levels [6,7]. African mothers replace themselves with nearly two daughters at a replacement fertility level of 2.1, which leads to rapid population growth [6].

Ethiopia has experienced high and persistent fertility rates for a long time. The TFR has declined from 5.5 children per woman in 2000 to 5.4 children per woman in 2005 to 4.8 children per woman in 2011 and to 4.6 children per woman in 2016 [8,9]. The fertility rate of Ethiopia is still high as compared to developed nations, even though the trend is declining [9]. The levels of maternal mortality and morbidity in Ethiopia are also among the highest in the world [9]. Short birth intervals, low maternal health service access, and high fertility seriously affect the health of mothers and children. Recent estimates showed that the country still experiences higher rates of maternal mortality, under-five mortality, and infant mortality of 412 deaths per 100,000, 67 deaths per 1000 live births, and 48 deaths per 1000 live births, respectively [9,10].

Of the Ethiopian population, more than 80% live in rural areas, whereas the high fertility and rapid population growth rate, especially in the rural areas, are unacceptably high, with a total fertility rate of above six children per woman [11]. In most Ethiopian rural communities, having a large family is still regarded as a source of pride and a divine gift [12]. Early age at first marriage, low socioeconomic status, culture, husband preferences, place of residence, region, education, and media exposure were significant factors leading to high fertility status [2,7]. High fertility status is also affected by the low level of health awareness and lack of access to modern contraceptives, especially in most of sub-Saharan Africa [2].

There is a highly skewed distribution of high fertility among reproductive-age women across socioeconomic, obstetric, and geographical lines. Ethiopia has not yet conducted spatial analyses to identify areas with high fertility among reproductive-age women. Moreover, information regarding the magnitude and associated factors of high fertility among reproductive-age women in Ethiopia remains unclear. Understanding the level and geographical variation of high fertility status in Ethiopia can help health planners, programmers, partners in the health sector, and policymakers formulate appropriate strategies and interventions and provide quality reproductive health services to reduce high fertility. Hence, this study aimed to assess the spatial distribution of high fertility status and associated factors in Ethiopia.

## Methods

In Ethiopia, there are nine regions (Afar, Tigray, Amhara, Oromia, Somali, Southern Nations, Nationalities, and People’s Region (SNNPR), Benishangul Gumuz, Gambella, and Harari) and two administrative cities (Addis Ababa and Dire Dawa) [13]. Based on Worldometer’s analysis of the latest United Nations data, Ethiopia has 121,989,792 citizens as of Thursday, December 22, 2022 [14].

Secondary data analysis was done based on the 2016 Ethiopian Demographic and Health Survey (EDHS), which was a national representative sample conducted from January 18, to June 27, 2016. The EDHS 2016 was accessed from the DHS official database, www.measuredhs.com, after permission was secured through an online request by explaining the purpose of the study, which were used a cross-sectional study design and a two-stage stratified cluster sampling technique to select populations using the 2007 Population and Housing Census (PHC) as a sampling frame. Stratification was done by separating the nine regional states and the two city administrations of Ethiopia, into urban and rural areas [13].

A total of 645 Enumeration Areas (EAs) (202 in urban areas and 443 in rural areas) proportional to EA size were selected proportionally to the EA size in the first stage. In the second stage, 28 households in each cluster were selected with an equal probability of systematic selection [9]. For this study, the study population was all women of the reproductive age group from 25 to 49 years. this group of women was selected for this study by considering the fact that women in the study area who have given birth at early age have the possibility of giving birth five and more children before they celebrate their twenty fifth birthday [7]. We used individual datasets. A **total weighted sample of 9398 reproductive age women were included in the present study.** Additionally, latitude and longitude coordinates were taken from selected EAs (clusters). Full details of the EDHS sampling system were presented in the report [13,15].

### Study variables

The outcome variable was fertility status measured by the number of children ever born alive. It is categorized as high fertility when the number of children ever born alive is ≥ 5 and low fertility when the number of children ever born alive is < 5. The cut-off point of 5 is taken because the medical and obstetric risk for women with the number of children ever born alive greater or equal to 5 is significantly higher compared with that of less than five [1,2,7,15].

Depending on different literature reviews, individual and community-level variables were included in the analysis. Women’s education (no formal education, primary education, and secondary education and above), religion (Orthodox, Muslim, and Protestant), contraceptive methods use (not using any methods, short acting family planning, and long acting family planning), age at first sex (< 18 years, ≥18 years), age at first birth (< 18 years, ≥18 years), unmet need for family planning (unmet need, met need, and infecund/menopausal) were considered as individual-level variables. Media exposure; those who read newspapers, listened to the radio, or watched television at least once a week were coded yes and no otherwise [16]. The household wealth index was calculated using consumer goods like televisions, bicycles, and cars. Materials used for the roof, floor, and toilet facilities were considered in calculating the household wealth index. To categorize individuals into wealth quintiles (poor, meddle, and rich), we used household asset data via principal component analysis (PCA) [17].

Of the community-level factors, distance to the health facilities (big problem, not big problems), and residence (rural, urban) were directly accessed from the EDHS data set. However, the aggregate community-level independent variables (community-level media exposure and community-level education) were constructed by aggregating individual-level characteristics at the community (cluster) level. They were categorized as high or low based on the distribution of the proportion values computed for each community after checking the distribution by using the histogram. The aggregate variable was not normally distributed, and the median value was used as a cut-off point for the categorization [18–20].

### Data management and analysis

For data analysis, we used STATA 14, ArcGIS 10.8, and SaTScan 9.6 software. For the analysis, sample weights were applied to adjust for the non-proportional sampling of strata and regions during the survey process and to restore representativeness. Text, figures, and tables were used to present descriptive statistics and summary statistics [13,20].

### Spatial analysis

#### Spatial autocorrelation analysis

The presence of spatial autocorrelation was identified using Moran’s index (Moran’s I). A Moran’s I value close to -1 indicates that disease/events are dispersed, whereas a Moran’s I value close to +1 indicates that they are clustered, and a Moran’s I value of zero indicates that they are distributed randomly. There was a significant Moran’s I (p < 0.05), indicating the presence of spatial autocorrelation and rejecting the null hypothesis (high fertility is randomly distributed). Hotspot analysis was conducted using the Getis-Ord Gi* statistic [13].

#### Spatial scan statistical analysis

Spatial scan statistics applied using Kulldorff’s SaTScan software identified statistically significant primary (most likely) and secondary clusters of high fertility status. In SaTScan works, a window moves across the study areas and the window size needs to be fixed. As the outcome variable was Bernoulli distribution, Kulldorff’s method was applieyd to use a Bernoulli model for a purely spatial analysis. In order to fit the Bernoulli model, respondents with high fertility were considered case, and those with low fertility were considered control. Using the default maximum spatial cluster size of 50% of the population as an upper limit, both small and large clusters were detected, and clusters with more than the maximum level were ignored. High fertility was considered in areas with a high Log Likelihood Ratio and significant p-value compared to areas outside the window.

#### Multi-level analysis

In the EDHS data, there was a hierarchical structure, which violates the independent observations and equal variance assumptions of a traditional logistic regression model. Therefore, women were nested within households, and households were nested within clusters. Within the cluster, they may have similar characteristics. Hence, multilevel binary logistic regression analysis must take into account the variability between clusters. Before adjusting for the variance through series of model development, we checked each variable at 0.2 p-values to include in the model. The final p-value remained <0.05 for the final model cut-point and AOR with 95% CI was also applied. Intra-class correlation coefficient (ICC), Median Odds Ratio (MOR), and Proportional Change in Variance (PCV) were computed to measure the variation between clusters. Taking clusters as a random variable, the MOR is defined as the median value of the odds ratio between the area at the highest risk and the area at the lowest risk area when randomly picking out two clusters. ${{MOR=e}^{0.95}}^{\sqrt{VA}}$ Whereas, the ICC reveals the variation of high fertility between clusters is calculated as; $ICC=\frac{VA}{VA+3.29}*100\%$. Moreover, the PCV reveals the variation in the high fertility among reproductive-age women explained by factors and calculated as; $PCV=\frac{Vnull-VA}{Vnull}*100\%$ where; Vnull = variance of the initial model, and VA = area/cluster level variance [21–23]. We applied deviance (-2LLR) to compare models. The lower the deviance the more fitted the model.

### Ethical approval and consent to participate

Written informed consent was waived from the International Review Board of Demographic and Health Surveys (DHS) program data archivists after the consent manuscript was submitted to the DHS program/ICF to download the dataset for this study. The study is not an experimental study. All the methods were conducted according to the Helsinki Declarations. More details regarding DHS data and ethical standards are available online at http://www.dhsprogram.com.

## Results

### Socio-demographic related factors

A total weighted sample of 9398 women were included in this analysis. The median age of the study participants was 33 years (IQR: 28–39). About 31.43% of participants fell within the age category of 25–29 years, and 78.89% of the women were rural dwellers. Majority (65.52%) of the participants had no formal education. About 45.52% of participants were orthodox religious followers (Table 1).

**Table 1:** Socio-demographic related factors of the participants of high fertility among reproductive age women in Ethiopia.

Variables	Frequency	Percentage
Region		
Tigray	619	6.59
Afar	71	0.76
Amhara	2318	24.66
Oromia	3399	36.16
Somali	271	2.88
Benishangul Gumuz	93	0.99
SNNPRs	2005	21.33
Gambela	25	0.27
Harari	22	0.24
Addis Ababa	523	5.56
Dire Dawa	52	0.56
Residence		
Rural	7414	78.89
Urban	1984	21.11
Age of the women		
25–29	2954	31.43
30–34	2333	24.82
35–39	1918	20.41
40–44	1252	13.32
45–49	942	10.02
Education of the women		
No formal education	6158	65.52
Primary education	2133	22.70
Secondary education and above	1107	11.78
Wealth index		
Poor	3355	35.69
Middle	1842	19.60
Rich	4202	44.71
Media exposure to family planning messages		
Yes	3730	39.69
No	5668	60.31
Distance to the health facilities		
Big problem	4847	51.57
Not big problem	4551	48.43
Religion		
Orthodox	4278	45.52
Muslim	2966	31.56
Protestant	2155	22.93
Community level education		
High	4435	47.19
Low	4963	52.81
Community level media exposure to family planning messages		
High	4725	50.28
Low	4673	49.72

### Obstetric related factors

About 68.17% and 42.22% of the women did not use any form of family planning methods and gave birth at home, respectively. More than two thirds (66.94) of the respondents had sex before 18 years old (Table 2).

**Table 2:** Obstetric-related factors of the participants of high fertility among reproductive age women in Ethiopia.

Variables	Frequency	Percentages
Place of delivery		
Home	3968	42.22
Health institution	5430	57.78
Age at first sex		
< 18 years	6291	66.94
≥ 18 years	3106	33.06
Age at first birth		
< 18 years	3532	37.58
≤ 18 years	5866	62.42
Contraceptive methods use		
Not using any methods	6407	68.17
Short acting family planning	2102	22.36
Long acting family planning	890	9.47
Unmet need for family planning		
Unmet need	2394	25.47
Met need	5217	55,51
Infecund/menopausal	1788	19.02

### Regional proportion of high fertility status among reproductive age women in Ethiopia

**Fig 1:**
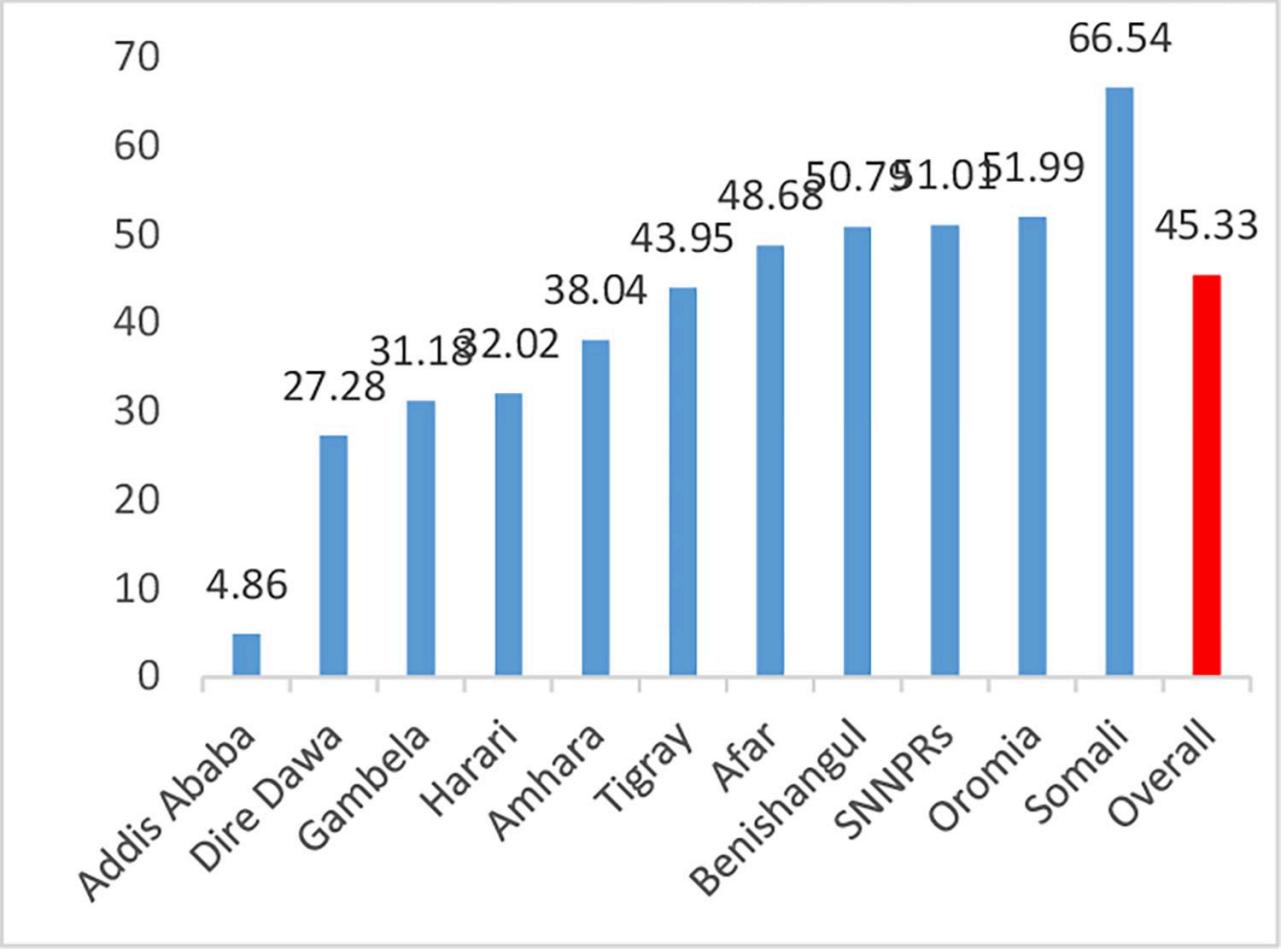
Regional proportion of high fertility status among reproductive age women in Ethiopia.

The proportion of high fertility varies across the country. In this study, about 45.33% (confidence interval: (44.32, 46.33) of reproductive-ag women had high fertility. The highest and lowest proportion of high fertility were observed in the Somali region (66.54%) and Addis Ababa (4.86%), respectively (Fig 1).

### Spatial analysis of high fertility status

#### Spatial autocorrelation and spatial analysis of high fertility status

**Fig 2:**
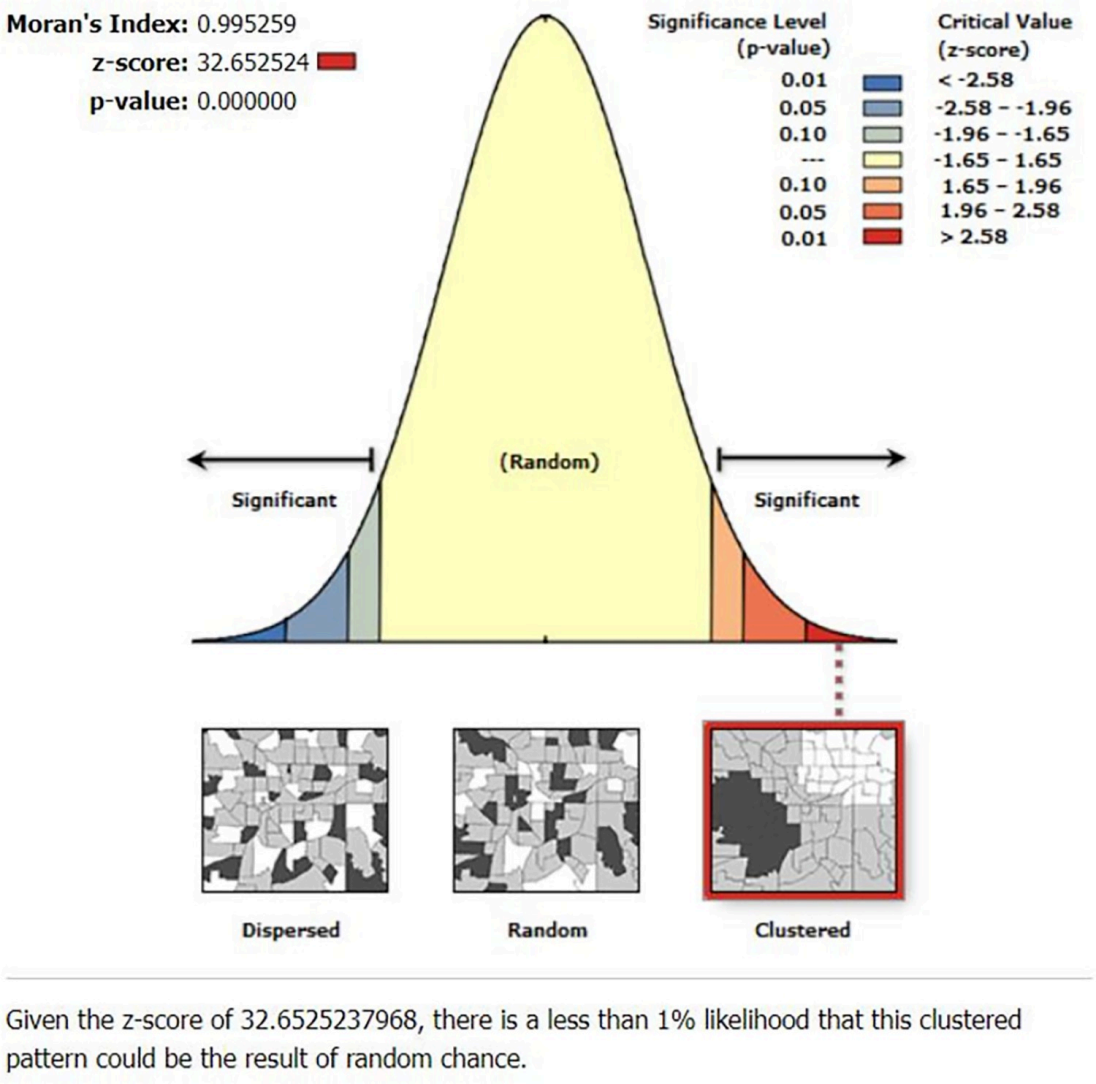
Spatial autocorrelation analysis of high fertility status among reproductive age women in Ethiopia.

**Fig 3:**
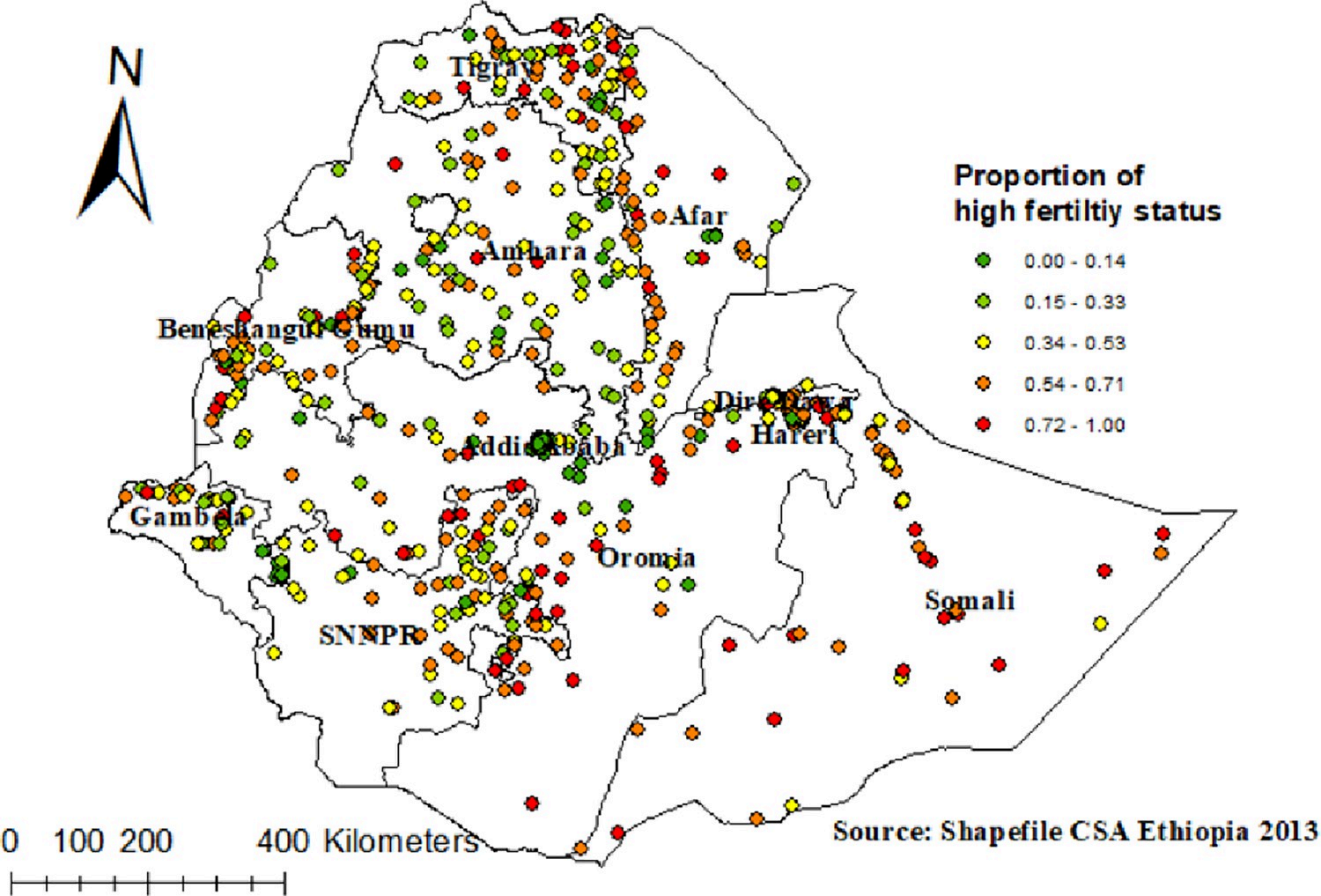
Spatial distribution of high fertility status among reproductive age women in Ethiopia, Shape file source: Central Statistical Agency 2013, URL: https://africaopendata.org/dataset/ethiopia shape files. Map output: Own analysis using ArcGIS 10.8 software.

The spatial autocorrelation analysis revealed that the distribution of high fertility was non-random in Ethiopia, with a Global Moran’s Index value of 0.99 (p<0.0001) (Fig 2). A higher proportion of fertility occurred in Somali region, Southeastern part of Oromia region and Northeastern part of SNNPR while low proportions of fertility were identified in the Addis Ababa, Harari, and Dire Dawa (Fig 3).

#### Getis OrdGi statistical analysis of high fertility

**Fig 4:**
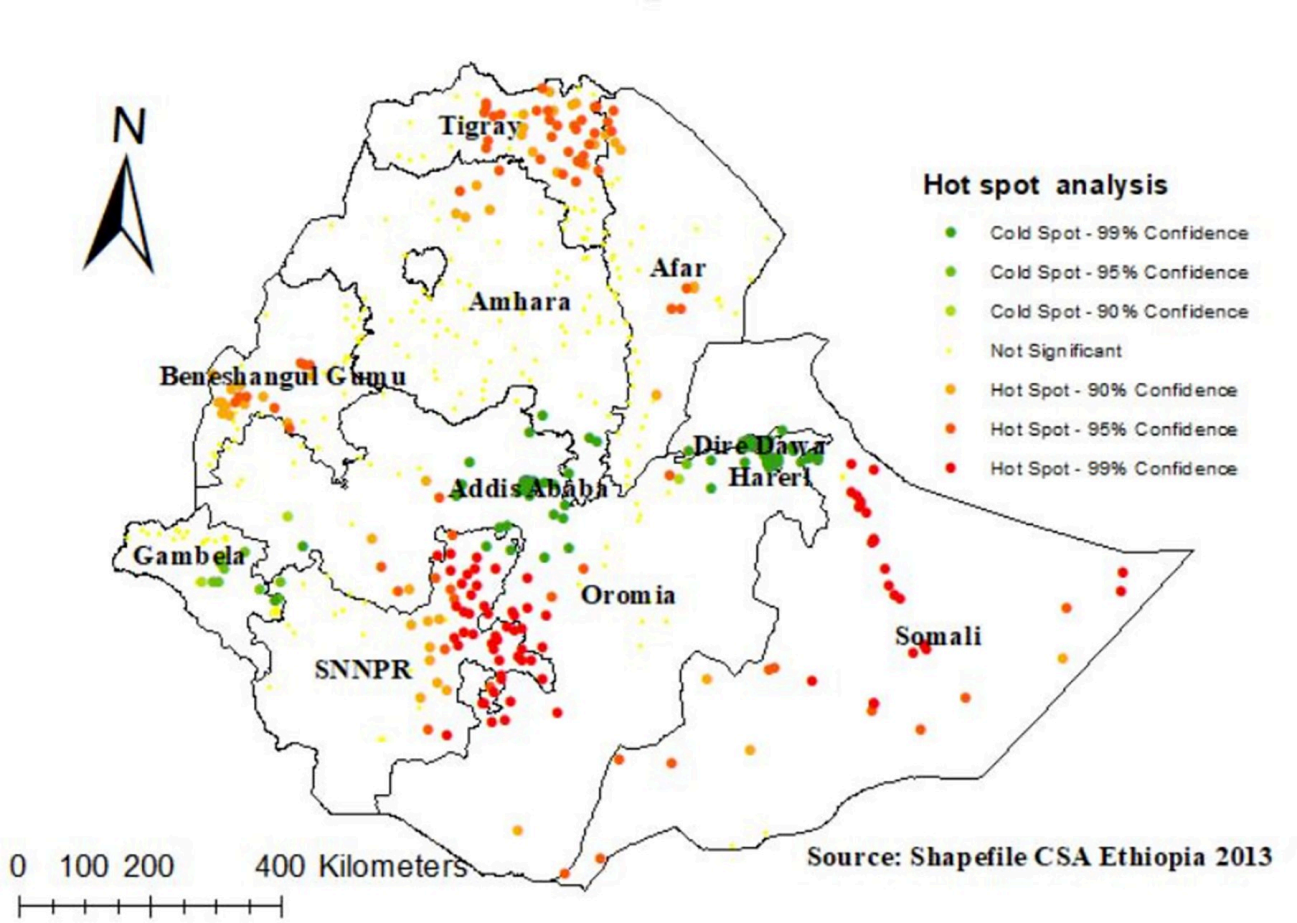
Hot spot analysis of high fertility status among reproductive age women in Ethiopia, Shape file source: Central Statistical Agency 2013, URL: https://africaopendata.org/dataset/ethiopia shape files. Map output: Own analysis using ArcGIS 10.8 software.

In the Getis OrdGi statistical analysis, significant hotspot areas (areas where fertility were high) were located in Somali, Central Afar, Northeastern part of SNNPR, and Southeastern part of Oromia region. Whereas the significant cold spot areas (areas with low fertility) were located in Addis Ababa, Dire Dawa, and Hareri (Fig 4).

#### Kriging interpolation

**Fig 5:**
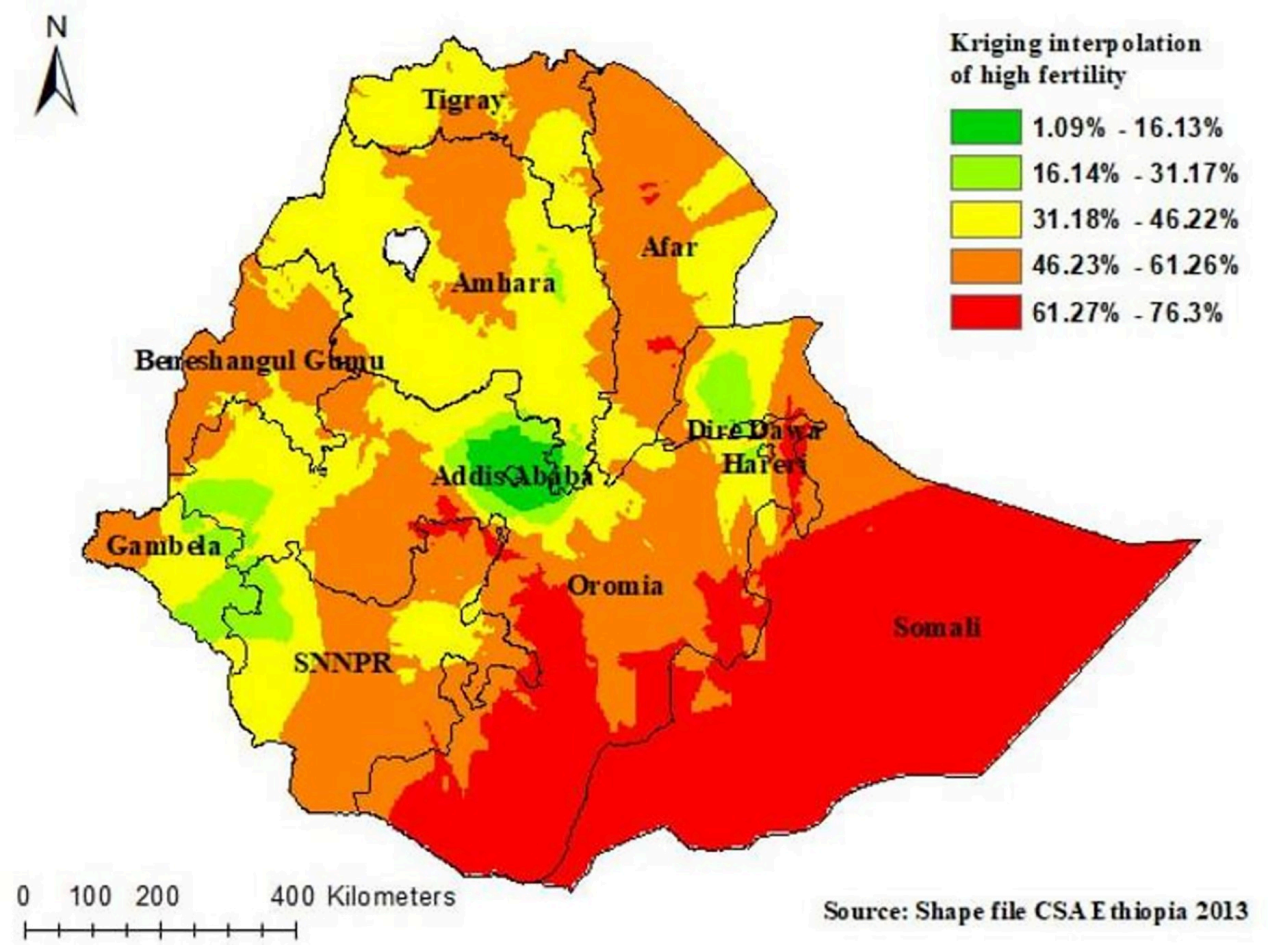
Kriging interpolation of high fertility status among reproductive age women in Ethiopia, Shape file source: Central Statistical Agency 2013, URL: https://africaopendata.org/dataset/ethiopia shape files. Map output: Own analysis using ArcGIS 10.8 software.

In the Kriging interpolation; the predicted high fertility status were identified in Somali and South and eastern parts of the Oromia region whereas, the predicted low fertility status was identified in the Addis Ababa, Dire Dawa, and Hareri (Fig 5).

#### Spatial Sa Tscan analysis of high fertility

**Fig 6:**
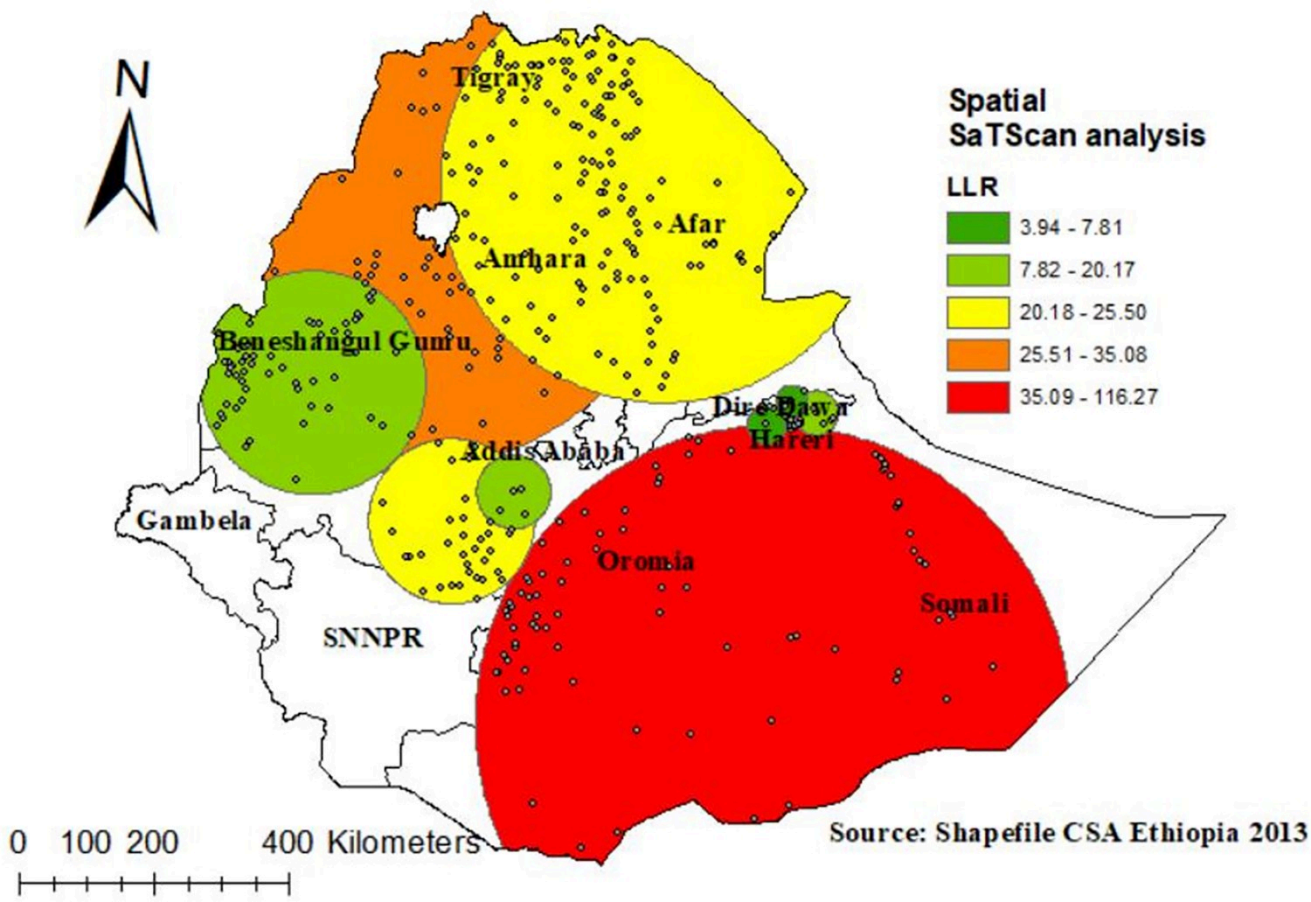
SaTScan analysis of high fertility status among reproductive age women in Ethiopia, Shape file source: Central Statistical Agency 2013, URL: https://africaopendata.org/dataset/ethiopia shape files. Map output: Own analysis using ArcGIS 10.8 software and SaTScan 9.6 software.

There were 565 significant clusters identified in the spatial Sa Tscan statistics, of which 86 were primary clusters (most likely). As a result of the survey, the primary clusters were found in the Somali, and eastern part of Oromia region. They were located at 5.330795 N, 41.837597 E of geographic location, with a radius of 440.63 km, with a Relative Risk (RR) of 1.6 and Log-Likelihood ratio (LLR) of 116.27, with p =  0.00001. According to the study, reproductive age women within the spatial window were 1.6 times more likely to have high fertility than reproductive age women outside it (Fig 6).

### Associated factors of TT immunization

#### Random effect results

The ICC value in the null model was 26.2% indicated that 26.2% of the total variability for high fertility as attributable to the between group variation while the remaining 73.8% was explained by the between individual variation. Besides, the MOR was 2.1 indicated that, if we randomly select two women from two different clusters, women at the cluster with a higher risk of high fertility had 2.1 times higher likelihood of high fertility compared with women at cluster with a lower risk of high fertility (Table 3).

**Table 3:** Multi-level mixed-effect logistic regression analysis of predictors of high fertility reproductive age women in Ethiopia.

Variables	Null model	Model I	Model II	Model III
Education of the mother				
No formal education		17. 49 (12.41, 24.67)		13.12 (9.27, 18.58)
Primary education		6.83(4.84, 9.65)		5.51 (3.88, 7.79)
Secondary education and above		1		1
Religion of the respondents				
Muslim		1.61 (1.35, 1.92)		1.52(1.28, 1.81)
Protestant		1.63 (1.35, 1.97)		1.48(1.23, 1.78)
Orthodox		1		1
Wealth index				
Poor		0.92 (0.79, 1.07)		0.84 (0.73, 1.05)
Middle		0.94 (0.81, 1.10)		0.87 (0.76, 1.09)
Rich		1		1
Unmet need for family planning				
Unmet need		1.55 (1.32, 1.81)		1.23 (0.97, 1;75)
Met need		0.95 (0.81, 1.12)		1.51 (0.75, 1.04)
Fecund/menopausal		1		1
Age at firs birth				
< 18 years		2.89 (2.57, 3.25)		2.94 (2.61, 3.31)
≥ 18 years		1		1
Contraceptive methods use				
Not using any methods		1.19 (		1.13 (0.92, 1.38)
Short acting family planning		1.26 (0.97, 1.46)		1.22 (0.98, 1.49)
Long acting family planning		1.23 (1.02 1.54)		1.19 (0.96, 1.48)
Age at first sex				
< 18 years		1.75 (1.53, 1.98)		1.70 (1.49, 1.93)
≥ 18 years		1		1
Media exposure to family planning message				
Yes		1		1
No		1.13(0.99, 1.28)		0.98 (0.87, 1.12)
Residence				
Rural			6.95 (5.39, 8.94)	3.76 (2.85, 4.94)
Urban			1	1
Distance to the health facilities				
Big problem			1.15 (1.03, 1.28)	1.19 (1.06, 1.34)
Not a big problem			1	1
Community media exposure				
Low			1.57 (0.94, 2.63)	1.58 (0.94, 2.66)
High			1	1
Community level education				
Low			0.91 (0.54, 1.52)	0.82 (0.49, 1.40)
High			1	1
Random effect result				
ICC	26.2	14.8	12.6	12.3
Variance	61.3	57.2	47.6	46.1
MOR	2.1	2.05	1.9	1.8
PCV (%)		6.7	22.4	24.8
Model fitness				
LL	-5828.8	-5034.6	-5648.3	-4958.4
Deviance	11657.6	10069.2	11296.6	9916.8

#### Fixed effect results

In the multivariable multilevel logistic regression analysis; women’s education, residence, religion, age at first sex, and age at first birth were predictors of high fertility in Ethiopia.

Accordingly, the odds of high fertility among women who had no formal education and had primary education were 13.12 (aOR: 13.12, 95% CI: 9.27, 18.58) and 5.51 (aOR: 5.51, 95% CI: 3.88, 7.79) times than those who had secondary education and above, respectively. Women who follow Muslim and protestant religion had 1.52 (aOR: 1.52, 95% CI: 1.28, 1.81) and 1.48 (aOR: 1.48, 95% CI: 1.23, 1.78) times more odds to have high fertility than those who follow orthodox religion. The odds of high fertility among women who gave birth first child before 18 years old (aOR: 2.94, 95% CI: 2.61, 3.31) were higher than those of women who gave birth the first child after 18 years old. The odds of high fertility among women who had their first sex before the age of 18 years old were 1.70 (aOR: 1.70, 95% CI: 1.49, 1.93) times higher than their counterparts. Moreover, women who lived in rural areas were 3.76 (aOR: 3.76, 95% CI: 2.85, 4.94) times more likely to have high fertility as compared with their counterparts (Table 3).

## Discussion

According to the present study, the spatial distribution of high fertility among reproductive-age women in Ethiopia was clustered. Significant hotspot areas with high fertility were identified in Somali, Central Afar, North-eastern part of SNNPR, and South-eastern part of Oromia region. Whereas the significant cold spot areas with low fertility were identified in Addis Ababa, Dire Dawa, and Harari. The possible justification for this difference could be due to the different socio-economic and obstetric-related factors of the study participants. For instance, the majority of the respondents in Addis Ababa, Dire Dawa, and Harari were educated, accessible to reproductive health services like family planning, and had a good awareness of reproductive health services [24,25]. In addition, most participants who lived in these regions were exposed to the media. Which in turn enhances the reproductive age women’s awareness on the effects of high fertility.

In the current study, residency was significantly associated with high fertility status. Women who reside in rural area have more children as compared to their counterparts. This is supported by studies done in Butajira [26], Ghana [27], and Nepal [28]. The possible justification might be that those who live in the rural area no longer stay in school, thereby married at school age [29]. Moreover, the demand for human power in agrarian living condition and the notion of considering the family with a large number of children as a blessed family in the rural area may drive couples to have an increased number of children. Moreover, access to media, general health knowledge, and better health service information, mainly characterizes urban women [30,31].

Another factor that affect the high fertility status found in our study was religion. Women who follow Muslim and protestant religions were more likely to have high fertility as compared with those who follow the orthodox religion. The possible reason might be the high proportion of Muslims (Afar, Somali, and Harar) and regions with a high proportion of Ethiopian Orthodox Christians (Addis Ababa, Amhara, and Tigray); fertility is much higher in developing regions like Somali; and most of the women in these regions have no formal education [9].

The odds of high fertility was higher among women with no formal education as compared to those with secondary education and above. This is in line with studies conducted in the Sidama region, Ethiopia [15], Nigeria [32], and Kenya [33]. The possible reason could be that women that are more educated may learn different ideas of desired family size through school, community, and exposure to global communication networks and know more about the risks of early birth and a short birth interval [34]. Moreover, educated women also know more about child health. Education generally results in an improvement in the status of women in society through a better understanding of health issues and employment status [33].

The likelihood of high fertility among women who had their first sex before 18 years old was higher than among those who had their first sex after 18 years. This could be because sex at an early age is a leading obstetric characteristic that indicates the exposure of women to teenage pregnancy [35]. This study also showed that high fertility was also higher among women who had their first birth before the age of 18 as compared to their counterparts. This is similar to the studies conducted in the Gedeo Zone, Ethiopia [7], and Nigeria [32]. This could be because women who started birth before 18 years, the period of fertility is longer, and they have many ever-born children. Usually, those women who started giving birth before the age of 18 resided in rural areas and had no formal education. As a result, they had poor knowledge of and access to reproductive health services like family planning methods [36].

The main strength of the current study was the use of weighted data that were nationally representative. As a result, the results of the current study can be applied nationally. In addition, we also identify similar and statistically significant areas with a high cluster of high fertility using both ArcGIS and Sat Scan statistical tests. As the study is cross-sectional in nature, a cause-and-effect relationship cannot be established. Moreover, due to the data being quantitative in nature, there are some hidden factors that need to be addressed by a qualitative study, and there might be biases like age at first birth and age at first sex.

## Conclusion

The spatial pattern of high fertility in Ethiopia is clustered. The significant hotspot areas (areas where fertility were high) were identified in Somali, Central Afar, the northeastern part of SNNPR, and the southeastern part of the Oromia region. Women’s education, residence, religion, age at first sex, and age at first birth were predictors of high fertility in Ethiopia. Therefore, designing a hotspot area-based interventional plan could help reduce high fertility. Moreover, much needs to be done among rural residents, including reducing early sexual initiations and the early age at first birth and enhancing women’s education. All the concerned bodies, including the kebele administration, religious leaders, and community leaders, should be in a position to ensure the practicability of the legal age of marriage.

## References

[1] Njoku CO, Abeshi SE, Emechebe CI: Grand Multiparity: obstetric outcome in comparison with multiparous women in a developing country. Open Journal of Obstetrics and Gynecology 2017, 7(07):707.

[2] Ajong AB, Agbor VN, Simo LP, Noubiap JJ, Njim T: Grand multiparity in rural Cameroon: prevalence and adverse maternal and fetal delivery outcomes. BMC pregnancy and childbirth 2019, 19(1):1–7. 31277596 10.1186/s12884-019-2370-z PMC6612095

[3] Bank W: Determinants and consequences of high fertility: a synopsis of the evidence: World Bank; 2010.

[4] Murray D: Maternal Mortality Rate, Causes, and Prevention. Verywell Family 2019.

[5] Johnson K, Abderrahim N, Rutstein SO: Changes in the direct and indirect determinants of fertility in sub-Saharan Africa: ICF Macro; 2011.

[6] Stover J, Brown T, Puckett R, Peerapatanapokin W: Updates to the Spectrum/Estimations and Projections Package model for estimating trends and current values for key HIV indicators. Aids 2017, 31(1):S5–S11. 28296796 10.1097/QAD.0000000000001322

[7] Reda MG, Bune GT, Shaka MF: Epidemiology of high fertility status among women of reproductive age in Wonago district, Gedeo Zone, Southern Ethiopia: a community-based cross-sectional study. International Journal of Reproductive Medicine 2020, 2020. doi: 10.1155/2020/2915628 32523965 PMC7261335

[8] Organization WH: Trends in maternal mortality: 1990–2015: estimates from WHO, UNICEF, UNFPA, World Bank Group and the United Nations Population Division: World Health Organization; 2015.

[9] Ethiopia CSA, Macro O: Ethiopia demographic and health survey. Addis Ababa: Central Statistical Agency 2016.

[10] Laelago T, Habtu Y, Yohannes S: Proximate determinants of fertility in Ethiopia; an application of revised Bongaarts model. Reproductive health 2019, 16(1):1–9. 30717804 10.1186/s12978-019-0677-x PMC6360651

[11] Ayele DG: Determinants of fertility in Ethiopia. African health sciences 2015, 15(2):546–551. doi: 10.4314/ahs.v15i2.29 26124801 PMC4480495

[12] Legesse A, Teferi B, Baudouin A: Indigenous agroforestry knowledge transmission and young people’s participation in agroforestry practices: The case of Wonago Woreda, Gedeo Zone, Southern Ethiopia. Acta Geographica-Trondheim Serie A 2013, 26:31.

[13] Tusa BS, Weldesenbet AB, Kebede SA: Spatial distribution and associated factors of underweight in Ethiopia: An analysis of Ethiopian demographic and health survey, 2016. Plos one 2020, 15(12):e0242744. doi: 10.1371/journal.pone.0242744 33259562 PMC7707465

[14] Woodward M, Reid MA: Cardiovascular disease in the Asia–Pacific region: challenges for health research and policy. The Medical Journal of Australia 2003, 179(2):71–72. doi: 10.5694/j.1326-5377.2003.tb05438.x 12864715

[15] Dasa TT, Okunlola MA, Dessie Y: Multilevel analysis of grand multiparity: Trend and its determinants in the Sidama National Regional State of Ethiopia: a cross-sectional study design from demographic and health survey 2000–2016. BMJ open 2022, 12(8):e061697. doi: 10.1136/bmjopen-2022-061697 35973699 PMC9386221

[16] Tadesse AW, Aychiluhm SB, Mare KU: Individual and community-level determinants of Iron-Folic Acid Intake for the recommended period among pregnant women in Ethiopia: A multilevel analysis. Heliyon 2021, 7(7):e07521. doi: 10.1016/j.heliyon.2021.e07521 34296017 PMC8282952

[17] Gebremedhin T, Aschalew AY, Tsehay CT, Dellie E, Atnafu A: Micronutrient intake status and associated factors among children aged 6–23 months in the emerging regions of Ethiopia: A multilevel analysis of the 2016 Ethiopia demographic and health survey. PloS one 2021, 16(10):e0258954. doi: 10.1371/journal.pone.0258954 34679088 PMC8535338

[18] Asmamaw DB, Eshetu HB, Negash WD: Individual and Community-Level Factors Associated With Intention to Use Contraceptives Among Reproductive Age Women in Sub-Saharan Africa. International Journal of Public Health 2022:107. doi: 10.3389/ijph.2022.1604905 35845431 PMC9278060

[19] Asratie MH, Andualem Z: Predictors of early resumption of post-partum sexual intercourse among post-partum period women in Ethiopia: A multilevel analysis based on Ethiopian demographic and health survey 2016. Plos one 2022, 17(9):e0271372. 10.1371/journal.pone.0271372 PMC9462818 36084107

[20] Asmamaw DB, Negash WD: Magnitude of unmet need for family planning and its predictors among reproductive age women in high fertility regions of Ethiopia: Evidence from Ethiopian Demographic and Health Survey. BMC Women’s Health 2022, 22(1):1–10. 36199076 10.1186/s12905-022-01982-w PMC9535900

[21] Liyew AM, Teshale AB: Individual and community level factors associated with anemia among lactating mothers in Ethiopia using data from Ethiopian demographic and health survey, 2016; a multilevel analysis. BMC Public Health 2020, 20:1–11. 32448212 10.1186/s12889-020-08934-9 PMC7247135

[22] Merlo J, Chaix B, Yang M, Lynch J, Råstam L: A brief conceptual tutorial of multilevel analysis in social epidemiology: linking the statistical concept of clustering to the idea of contextual phenomenon. Journal of Epidemiology & Community Health 2005, 59(6):443–449. 15911637 10.1136/jech.2004.023473 PMC1757045

[23] Merlo J, Chaix B, Yang M, Lynch J, Råstam L: A brief conceptual tutorial on multilevel analysis in social epidemiology: interpreting neighbourhood differences and the effect of neighbourhood characteristics on individual health. Journal of Epidemiology & Community Health 2005, 59(12):1022–1029. doi: 10.1136/jech.2004.028035 16286487 PMC1732971

[24] Kiross GT, Chojenta C, Barker D, Loxton D: Optimum maternal healthcare service utilization and infant mortality in Ethiopia. BMC Pregnancy and Childbirth 2021, 21(1):390. doi: 10.1186/s12884-021-03860-z 34011300 PMC8136182

[25] Yesuf KA, Liyew AD, Bezabih AK: Impact of Exposure to Mass Media on Utilization Modern Contraceptive Among Adolescent Married Women in Ethiopia: Evidence from EDHS 2016. Available at SSRN 3756795 2021.

[26] Mekonnen W, Worku A: Determinants of fertility in rural Ethiopia: the case of Butajira Demographic Surveillance System (DSS). BMC public health 2011, 11(1):1–6. doi: 10.1186/1471-2458-11-782 21985493 PMC3201928

[27] Odame ML: Delayed Marriage and Fertility Transition in Ghana. University of Ghana; 2018.

[28] Adhikari R: Demographic, socio-economic, and cultural factors affecting fertility differentials in Nepal. BMC pregnancy and childbirth 2010, 10(1):1–11. doi: 10.1186/1471-2393-10-19 20426863 PMC2885993

[29] Emirie G: Early Marriage and Its Effects on Girls’ Education in Rural Ethiopia: The Case of Mecha Woreda in West Gojjam, North-Western Ethiopia. 2005.

[30] Tarekegn SM, Lieberman LS, Giedraitis V: Determinants of maternal health service utilization in Ethiopia: analysis of the 2011 Ethiopian Demographic and Health Survey. BMC pregnancy and childbirth 2014, 14(1):1–13. doi: 10.1186/1471-2393-14-161 24886529 PMC4022978

[31] Tiruneh FN, Chuang K-Y, Chuang Y-C: Women’s autonomy and maternal healthcare service utilization in Ethiopia. BMC health services research 2017, 17(1):1–12. 29132363 10.1186/s12913-017-2670-9 PMC5683361

[32] Alaba OO, Olubusoye OE, Olaomi J: Spatial patterns and determinants of fertility levels among women of childbearing age in Nigeria. South African Family Practice 2017, 59(4):143–147.

[33] Mungai SW: Explaining high fertility in the north eastern region of Kenya. University of Nairobi; 2015.

[34] Pimentel J, Ansari U, Omer K, Gidado Y, Baba MC, Andersson N, Cockcroft A: Factors associated with short birth interval in low-and middle-income countries: a systematic review. BMC pregnancy and childbirth 2020, 20(1):1–17. doi: 10.1186/s12884-020-2852-z 32164598 PMC7069040

[35] Ariho P, Kabagenyi A: Age at first marriage, age at first sex, family size preferences, contraception and change in fertility among women in Uganda: analysis of the 2006–2016 period. BMC women’s health 2020, 20(1):1–13. 31948426 10.1186/s12905-020-0881-4 PMC6966849

[36] Ely DM, Hamilton BE: Trends in fertility and mother’s age at first birth among rural and metropolitan counties: United States, 2007–2017: US Department of Health and Human Services, Centers for Disease Control and …; 2018. 30475685

